# It is time to optimize forest management policy for both carbon sinks and wood harvest in China

**DOI:** 10.1093/nsr/nwae464

**Published:** 2024-12-19

**Authors:** Xi Li, Haicheng Zhang, Rong Shang, Jingmin Chen, Daju Wang, Jianhua Zhu, Huaguo Huang, Simei Lin, Baihong Pan, Wenping Yuan, Shilong Piao

**Affiliations:** Institute of Carbon Neutrality, Sino-French Institute for Earth System Science, College of Urban and Environmental Sciences, Peking University, China; School of Geography and Planning, Sun Yat-sen University, China; Key Laboratory for Humid Subtropical Eco-Geographical Processes of the Ministry of Education, School of Geographical Sciences, Fujian Normal University, China; Key Laboratory for Humid Subtropical Eco-Geographical Processes of the Ministry of Education, School of Geographical Sciences, Fujian Normal University, China; Department of Geography and Planning, University of Toronto, Canada; School of Atmospheric Sciences, Guangdong Province Data Center of Terrestrial and Marine Ecosystems Carbon Cycle, Sun Yat-sen University, China; Ecology and Nature Conservation Institute, Chinese Academy of Forestry, Key Laboratory of Forest Ecology and Environment of National Forestry and Grassland Administration, China; Key Laboratory for Silviculture and Conservation of Ministry of Education, Beijing Forestry University, China; Key Laboratory for Silviculture and Conservation of Ministry of Education, Beijing Forestry University, China; School of Biological Sciences, Center for Earth Observation and Modeling, University of Oklahoma, USA; Institute of Carbon Neutrality, Sino-French Institute for Earth System Science, College of Urban and Environmental Sciences, Peking University, China; Institute of Carbon Neutrality, Sino-French Institute for Earth System Science, College of Urban and Environmental Sciences, Peking University, China

Forestry protection has constituted a fundamental element of China's forest management policy for the past four decades, and has played a critical role in increasing forest area and biomass stock [1,2]. Nevertheless, an efficacious forest management policy should balance the dual roles of forests, serving as both carbon sinks and ecosystems [3], while also satisfying human demands for wood production. As one of the largest developing countries, China exhibited a significant increase in wood consumption from 49.42 million m^3^ in 1980 to 534.18 million m^3^ in 2020 [4]. However, the forest management policy in China tends to strictly limit domestic wood harvest for production. This forest management policy was formulated 40 years ago according to situations at that time. Here, we evaluate the continued reliability of this protection-based forest management policy in the context of significant changes in forest structure. Additionally, we proposed a new wood harvest scheme that can satisfy all domestic wood requirements and has no negative impacts on forest carbon sink and soil erosion.

Both the forest area and the stock in China have exhibited a notable increase over the past four decades. The forest area has almost doubled from 1.15 million km^2^ in 1980 to 2.20 million km^2^ in 2020 (Fig. 1a), representing the largest rate of increase in the world (Fig. S2a, b). Similarly, the forest stock in 2020 was 17.56 billion m^3^, ranking seventh globally (Fig. S1c, d). Furthermore, our findings demonstrated an expansion in forest area across various standard age groups, particularly in mature and near-mature forests (Fig. S3a, b). A comparison of the seventh (2004–2008) and the ninth (2014–2018) National Forest Inventories revealed an increase in the area of near-mature, mature and over-mature forests by 24.12%, 31.87% and 25.83%, respectively, suggesting a gradual enhancement in the forest's wood supply capacity.

**Figure 1.**
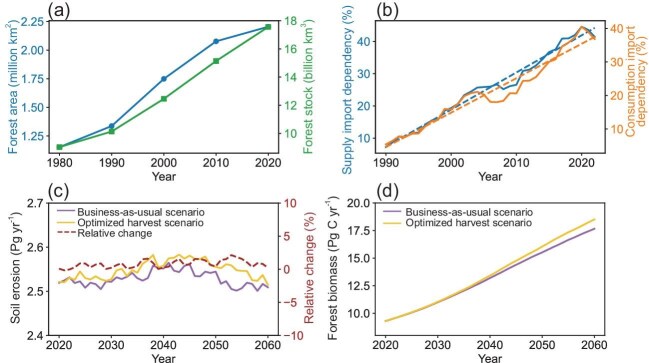
Long-term variations of forest area and forest stock (a), the supply import dependency and consumption import dependency (b), the simulated soil erosion under two wood harvest scenarios (The relative change refers to the ratio of the difference between optimized harvest scenario and business-as-usual scenario to business-as-usual scenario for each year) (c), and simulated forest biomass under the two scenarios (d).

Compared with the large increases of forest area and stock, the domestic wood harvest in China is quite limited. According to our analysis, the wood harvest intensity (defined as the ratio of harvested biomass to forest area) in China was 153 m³ km^−2^ in 2020, which was lower than the other 108 countries (Fig. S4). However, wood utilization in China significantly increased (5.20 million m^3^ yr^−1^) from 1990 to 2022 (Fig. S5). Consequently, China became one of the countries with the highest supply import dependency (42.55%) and consumption import dependency (40.12%) (Fig. S6), comparable to countries with limited domestic forest resources (e.g. Kazakhstan and Pakistan). Additionally, supply import dependency and consumption import dependency increased significantly by 1.04% yr^−1^ (*P* < 0.01) and 0.94% yr^−1^ (*P* < 0.01), respectively, from 1990 to 2022 (Fig. 1b). We further employed three risk indices, consisting of political risk, trade risk and resource risk, to explore the constraints and security of wood imports (section S1.2). All wood import risk indicators showed upward trends since 1990, i.e. trade risk (0.02 yr^−1^), political risk (0.03 yr^−1^) and resource risk (0.13 yr^−1^) (Fig. S7).

It had long been presumed that wood harvest would decrease forest carbon sink and result in emissions of stored carbon due to wood production and usage. In this study, we explored the impact of wood harvest from three aspects. Firstly, the impact of wood harvest on forest carbon sink was quantified under two harvest scenarios using a semi-empirical relationship between biomass and forest ages [5]. The business-as-usual (BAU) scenario is defined as maintaining the current annual wood harvest volume (i.e. 0.3 billion m^3^ yr^−1^ from mature and over-mature planted forests), with the remaining 0.2 billion m^3^ yr^−1^ being imported. The optimized harvest (OPT) scenario is defined as selectively harvesting 0.5 billion m^3^ yr^−1^ of mature and over-mature trees from both planted and natural forests, which would meet the annual domestic wood requirement (section S1.3). The mean annual harvest ratio of planted forest stock in the BAU scenario is 14.39%, with 85.61% of the stock retained. The mean annual harvest ratios of natural forest stock and planted forest stock are estimated to be ∼3.4% and ∼13.42%, respectively, in the OPT scenario, with the remaining portion retained. Studies of forest growth curves have demonstrated that carbon sequestration in young forests occurs rapidly and peaks at around 40 years of age [6,7]. The results of the model experiments demonstrated that the long-term trend of forest biomass from 2020 to 2060 in the OPT scenario was 0.225 Pg C yr^−1^ (Fig. 1d), which was ∼1.12 times compared with that of the BAU scenario (0.201 Pg C yr^−1^). Consequently, the OPT scenario is predicted to increase the forest carbon sink by ∼4.79% in 2060 (Fig. S5 and Fig. S9).

Secondly, the CO_2_ emissions resulting from wood products are highly dependent on the usage of the wood products following harvesting [8]. The estimation of CO_2_ emissions was based on the Long-term harvEst and Allocation of Forest Biomass (LEAF) dataset [9]. The results demonstrated that only 2.13% of harvested wood was utilized for paper and paperboard, which exhibited a rapid turnover rate (0.84 Mt C yr^−1^) from wood products to CO_2_. The majority of harvested wood (60.27%) was used for wood-based panels, constructions and furniture. These wood products were found to store carbon over extended periods (Fig. S8a). Once the wood products were out of their servers, they were discarded and entered landfills, where the decomposition is slow [10]. In total, from 2003 to 2100, on average, it is predicted that the discarded wood products released will be 3.93 Mt C yr^−1^ for wood-based panels, 0.71 Mt C yr^−1^ for furniture and 1.78 Mt C yr^−1^ for constructions (Fig. S8b). This suggests that the promotion of long-term cycle wood products could enhance carbon sequestration and extend carbon storage duration [2].

Additionally, we investigated the potential sediment loss due to forest harvesting under two forest harvest scenarios (section S1.5). The findings indicated that sediment loss under both the BAU and OPT scenarios increased for approximately the first 20 years and then decreased (Fig. 1c). The average sediment loss from 2020 to 2060 in the OPT scenario is 2.55 Pg yr^−1^, representing a 0.79% increase compared with the BAU scenario (i.e. 2.53 Pg yr^−1^; Fig. 1c). This reveals that the increased wood harvest resulted in only minor increases in soil erosion (Fig. S10).

This study highlights that it is time to change the current protection-oriented forest management policy in China, which has resulted in an insecure wood supply and decrease of forest carbon sink. The proposed wood harvest scheme is capable of satisfying all domestic wood requirements, thereby ensuring wood consumption security, and increasing the forest carbon sink by ∼3.16% averaged from 2020 to 2060, while only increasing soil erosion by 0.79%. In general, our study highlights that national forest management policy should transfer from protection-oriented to balance of usage and protection to achieve the win-win target for forest carbon sink and wood harvest in China. Simultaneously, it is crucial to consider the risks associated with the implementation of forest management policy. For instance, due to the complexity of implementation, selective cutting may be carried out as total cutting, significantly reducing forest productivity. Furthermore, landfills might be replaced with burning, resulting in immediate emissions of stored carbon in the wood products. Therefore, the implementation of forest management policy should be coordinated with various economic sectors (e.g. manufacturing and trash disposal) to jointly mitigate these risks.

## Supplementary Material

nwae464_Supplemental_File
