# Supplementary material for: It is time to optimize forest management policy for both carbon sinks and wood harvest in China
Source: Natl Sci Rev. 2024 Dec 19;12(4):nwae464. doi: 10.1093/nsr/nwae464 (PMC11887849; doi:10.1093/nsr/nwae464)
Supplement: nwae464_Supplemental_File [file nwae464_supplemental_file.docx]

Supplementary Materials for

**It is time to optimize forest management policy for both carbon sinks and wood harvest in China**

Xi Li^1^, Haicheng Zhang^2^, Rong Shang^3^, Jingmin Chen^3,4^, Daju Wang^5^, Jianhua Zhu^6^, Huaguo Huang^7^, Simei Lin^7^, Baihong Pan^8*^, Wenping Yuan^1^, Shilong Piao^1^

*Corresponding author: Baihong Pan, Email: baihong.pan@ou.edu

**This file includes:**

1. Data and methods

2. Figure S1 to S10

**1 Data and methods**

1.1 The wood import dependence

In this study, two indexes were used to indicate the extent of wood import dependence. The first index is the wood supply import dependence (*T_SID_*), which is defined as the proportion of imported wood resources (*I_Logs_*) to the sum of domestic wood production (*D_Logs_*) and imported wood resources (*I_Logs_*) [[1](#_ENREF_1), [2](#_ENREF_2)]. The equation used to calculate this is as follows:

$T_{SID}=\frac{I_{Logs}}{D_{Logs}+I_{Logs}}$ (1)

The second index is wood consumption import dependence (*T_CID_*), which refers to the proportion of net imported wood resources (imported wood resources minus exported wood resources) in relation to the total consumption of wood resources. This can be expressed as follows:

$T_{CID}=\frac{I_{Logs}-E_{Logs}}{D_{Logs}+I_{Logs}-E_{Logs}}$ (2)

where *E_Logs_* represents the quantity of exported wood resources.

All harvested wood products are traced back to their origin as roundwood. This process entails the conversion of the total quantity of imported (exported) harvested wood products into roundwood equivalents, with the use of roundwood equivalent coefficients [[1](#_ENREF_1)]. The roundwood equivalent coefficients were sourced from the National Forestry and Grassland Administration (<https://www.forestry.gov.cn/>) [[2](#_ENREF_2)].

1.2 The risk of wood import

In order to gain a more nuanced understanding of the security of wood supply, this study employed three risk indexes to quantify the security of wood import including political risk, trade risk, and resource risk [[3](#_ENREF_3)]. Political risk (PR) represents the most significant factor influencing imports, with the political conditions in source countries being of paramount importance for China's wood imports. This can be expressed as:

$PR=[\sum_{i} \left( \frac{{NPI}_{i}}{NPI} \right)^{2}{(100-WGI}_{i})\cdot p_{i}]\cdot s$ (3)

where *NPI_i_* represents the quantity of imported wood from the *i*th exporting country to China, while *NPI* represents the total quantity of wood imported to China. *WGI_i_* represents World Governance Indicators (WGI) (<https://data.worldbank.org/>), which quantifies the effectiveness of governance in different countries; *p_i_* denotes the proportion of wood exports to total wood production from each country, while *s* indicates China's wood consumption import dependence (section S1.1).

The level of trade risk (TR) is contingent upon the trade freedom of each exporting country, the distance between China and exporting countries, and the existence of a shared border.

$TR=[\sum_{i} \left( \frac{{NPI}_{i}}{NPI} \right)^{2}{(100-t}_{i})\cdot d_{i}\cdot b_{i}\cdot p_{i}]\cdot s$ (4)

where the distance *d_i_* between China and the *i*th exporting country is calculated as the ratio of the distance between capitals to the distance between the two countries. This data is sourced from the Centre for Prospective Studies and International Information (CEPII) (<https://www.cepii.fr/>). Additionally, *b_i_* is a binary variable that takes a value of 1 if the two countries share a border, and 2 otherwise. Finally, *t_i_* represents trade freedom. The data on trade freedom was obtained from the Heritage Foundation Database (<https://www.heritage.org/index>).

The resource risk (RR) is closely related to the forest stock volume in exporting countries.

$RR=[\sum_{i} \left( \frac{{NPI}_{i}}{NPI} \right)^{2}\cdot{PR}_{s}]\cdot s$ (5)

where *PR_s_* represents the ratio of wood production to forest stock volume in *i*th exporting country. Forest stock volume is derived from the Food and Agriculture Organization (FAO) of the United Nations (https://www.fao.org/faostat/).

1.3 Wood harvest scenarios

To ascertain the extent to which wood harvesting affects forest carbon sink and soil erosion, two distinct scenarios for wood harvesting over the subsequent 40 years, based on their respective contribution to domestic wood requirements, have been devised and presented in this study. It was assumed that the annual domestic wood requirement would remain constant at the current requirement level (i.e., 0.5 billion m³ yr^-1^; Fig. S5) over the subsequent 40 years. The first scenario is business-as-usual (BAU) scenario, which assumes that the annual wood harvest will maintain the current harvest volume, namely 0.3 billion m^3^ yr^-1^ from mature and over-mature planted forests. The remaining 0.2 billion m^3^ yr^-1^ will be imported, resulting in a wood consumption import dependency of approximately 40% (Fig. S6b). The second optimized harvest (OPT) scenario involves harvesting 0.5 billion m^3^ yr^-1^ of mature and over-mature trees from both planted and natural forests. Furthermore, natural forests are harvested only when planted sources are unable to meet demand. It is not permissible to harvest forests within natural reserves or on slopes exceeding 25 degrees. The harvest ratio of planted forest stocks is set between 0% to 50%, with harvesting carried out randomly in areas of mature and over-mature forests. In consideration of conservation policies pertaining to natural forests, the harvest volume for each grid of natural forest is limited to a maximum of 30%. Based on the final results of the future scenarios, the mean annual harvest ratio of planted forest stocks in the BAU scenario is 14.39%. In the OPT scenario, the mean annual harvest ratios of natural forest stocks and planted forest stocks are estimated to be approximately 3.4% and 13.42%, respectively.

1.4 Forest carbon sink estimates

The estimation of forest carbon sink under different wood harvest scenarios is conducted using the method developed by Shang et al. [[4](#_ENREF_4)]. Briefly, this method employs a semi-empirical model based on forest growth curves of diverse forest types. The annual increment of forest biomass (*∆biomass*) at a given stand age can be estimated as the following equation:

$\Delta biomass\left( p \right)=r[1+\frac{(g({\frac{p}{s})}^{d}-1)}{\exp\left( \frac{p}{s} \right)}]$ (6)

where *p* represents the forest age. The model parameters *r*, *g*, *s*, and *d* are estimated based on the measurements of a forest field survey conducted by Tang et al. [[5](#_ENREF_5)]. In addition, Shang et al. [[4](#_ENREF_4)] predicted future *∆biomass* based on estimated *∆biomass* from Eq. (6) and satellite-based biomass observations:

$\Delta biomass\left( j \right)={\Delta biomass}_{ref}\frac{f(age\left( j \right))}{f(age\left( 2020 \right))}$ (7)

where *age(j)* and *age(2020)* indicate the forest age at year of *j* and 2020. *f(age(j))* and *f(age(2020))* are the normalized *∆biomass* predicted by the Eq. (6). Normalization is achieved by dividing each value by the maximum *∆biomass* in the curve [4]. The *∆biomass_ref_* in 2020 is derived by averaging *∆biomass* from 2010 to 2020. Forest biomass data are sourced from the National Cryosphere Desert Data Center (<http://ncdc.ac.cn/portal/>). The forest biomass at year of *j* is calculated as the initial forest biomass in 2020 plus the cumulative *∆biomass* over *j* years. The carbon conversion factors provided by the IPCC [[6](#_ENREF_6)] were applied to convert the harvested volume (m³) to harvested biomass (Mg C).

1.5 Soil erosion simulations

In this study, the ORCHIDEE-LSM was employed for the simulation of soil erosion under two harvesting scenarios. The ORCHIDEE-LSM incorporates a hydrological module, namely SECHIBA (A Simple Parameterization of the Hydrologic Exchanges Between the Soil-Vegetation System and the Atmosphere) [[7](#_ENREF_7)], which is used to simulate the fluxes of energy and water at the atmosphere-land surface interface, encompassing the dynamics of soil water [[8](#_ENREF_8)]. We used climate predictions by an earth system model (i.e., CESM, Community Earth System Model) under the SSP4.5 to drive the model over the next 40 years. Other model forcing data and model scheme are introduced by Zhang et al. [[9](#_ENREF_9)].

1.6 Global forest resources assessment

The global forest resource assessment data is derived from the Food and Agriculture Organization (FAO) of the United Nations. The forest area, forest stock and harvested forest datasets for countries worldwide were extracted for the years 1990, 2000, 2010, and 2020. Further details regarding this dataset can be accessed via the FAO Global Forest Resources Assessment (https://fra-data.fao.org/assessments/).

1.7 Forestry production and trade data

The Food and Agriculture Organization (FAO) forestry production and trade (wood import and wood export) data for the period 1990 to 2022 (https://www.fao.org/faostat/) were employed to conduct a comprehensive examination of the variations in wood trade across different nations. The dataset encompasses nine wood products, including fiberboard, paper and paperboard, particle board, plywood, recovered paper, roundwood, sawn wood, wood charcoal, and wood pulp. The quantity of wood imported by China from each exporting country was obtained from the United Nations Commodity Trade Database (https://comtradeplus.un.org/).

1.8 National forest inventory (NFI) survey data

The National Forest Inventory (NFI) survey data were collected through nationwide systematic point sampling and plot surveys [[10](#_ENREF_10)]. In this study, we employed forest age-group data from the 7^th^ National Forest Inventory (2004-2008), the 8^th^ National Forest Inventory (2009-2013), and the 9^th^ National Forest Inventory (2014-2018) to examine the alterations in the forest age structure in China. The selected datasets encompass young forests, middle-aged forests, near-mature forests, mature forests, and over-mature forests.

1.9 China vegetation products dataset

The China Vegetation Products version 1.0 (CVP v1.0) (https://www.3decology.org/), encompassing the planted and natural forests maps in 2020 [[11](#_ENREF_11)], forest age map in 2020 [[12](#_ENREF_12)]. The forest biomass data is sourced from the China Forest Aboveground and Belowground Biomass Carbon Change Dataset (2002-2021) [[13](#_ENREF_13)].

1.10 The Long-term harvEst and Allocation of Forest Biomass (LEAF) dataset

The Long-term Harvest and Allocation of Forest Biomass (LEAF) dataset was used to investigate the accumulated carbon stock of harvested wood products and annual CO_2_ emissions in this study. The LEAF dataset was produced based on the statistical data of each province, which is appropriate for indicating emissions of wood products in China [[14](#_ENREF_14)].

2 Figure S1 to S10


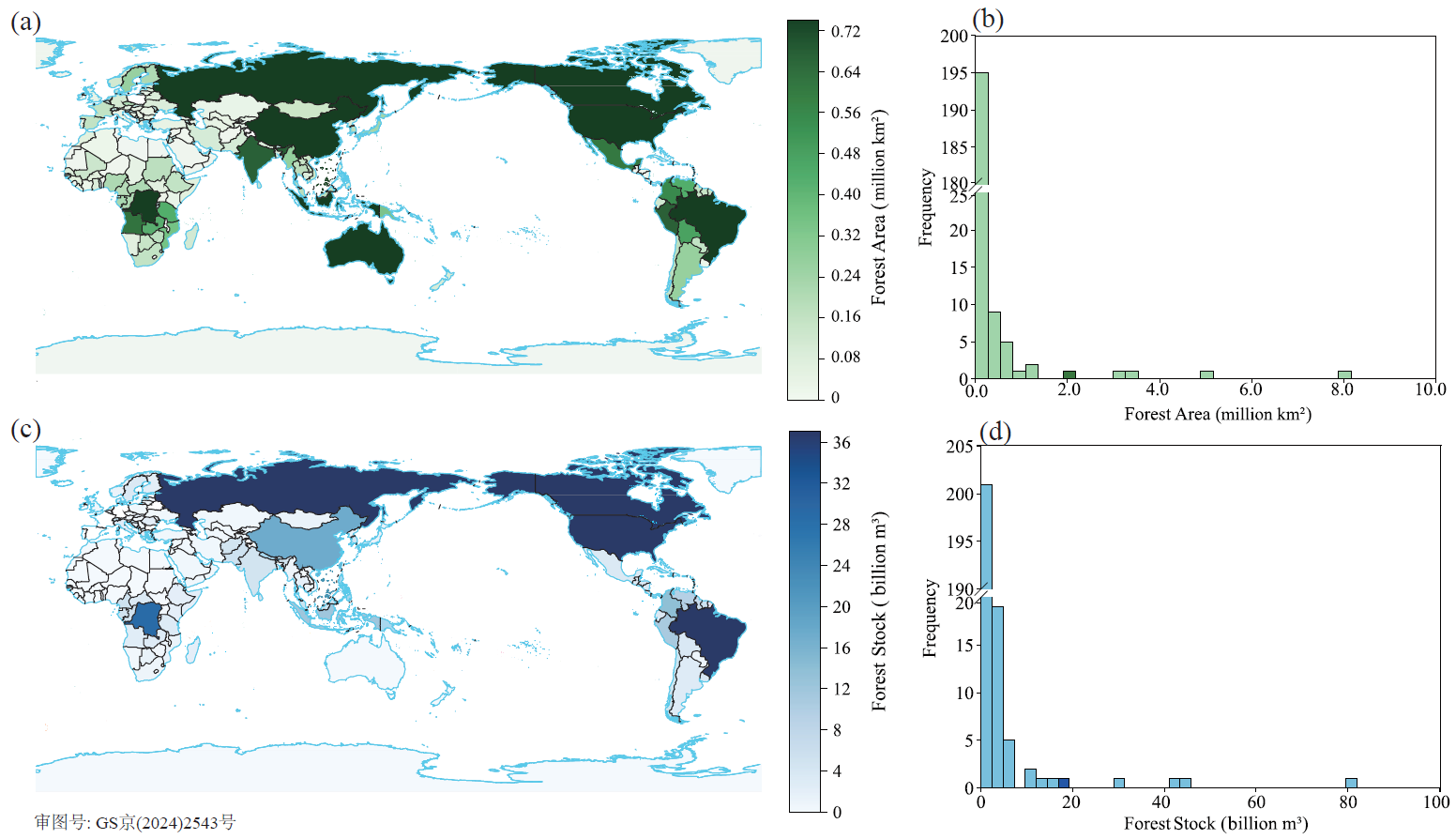


**Figure S1**. Global pattern of global forest area (a-b) and forest stock (c-d) in 2020. (a) and (c) show the spatial distribution and (b) and (d) show frequency histogram.


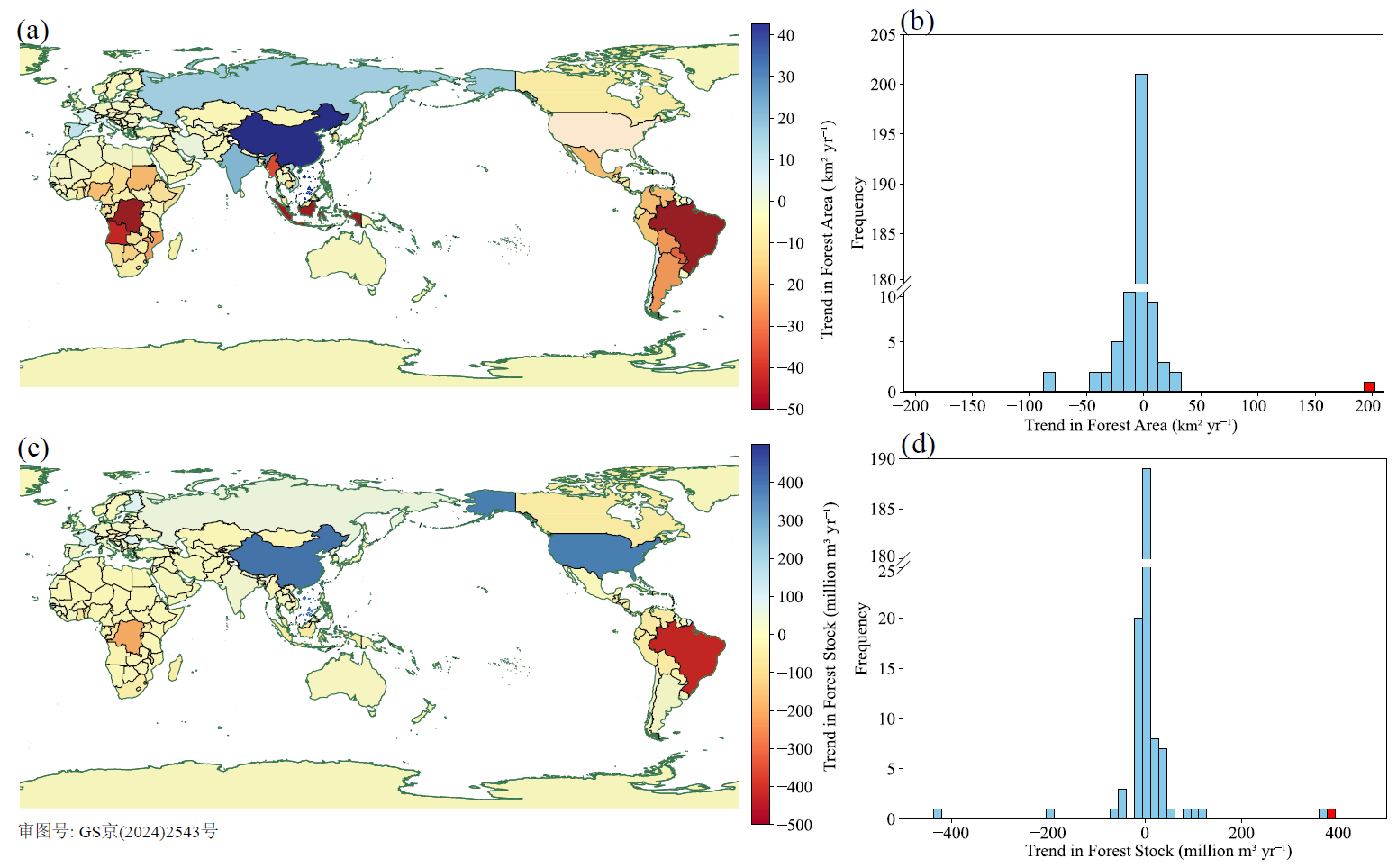


**Figure S2**. Global pattern on the long-term trends of forest area (a-b) and forest stock (c-d) from 1990 to 2020. (a) and (c) show global distribution. (b) and (d) show the frequency histogram.


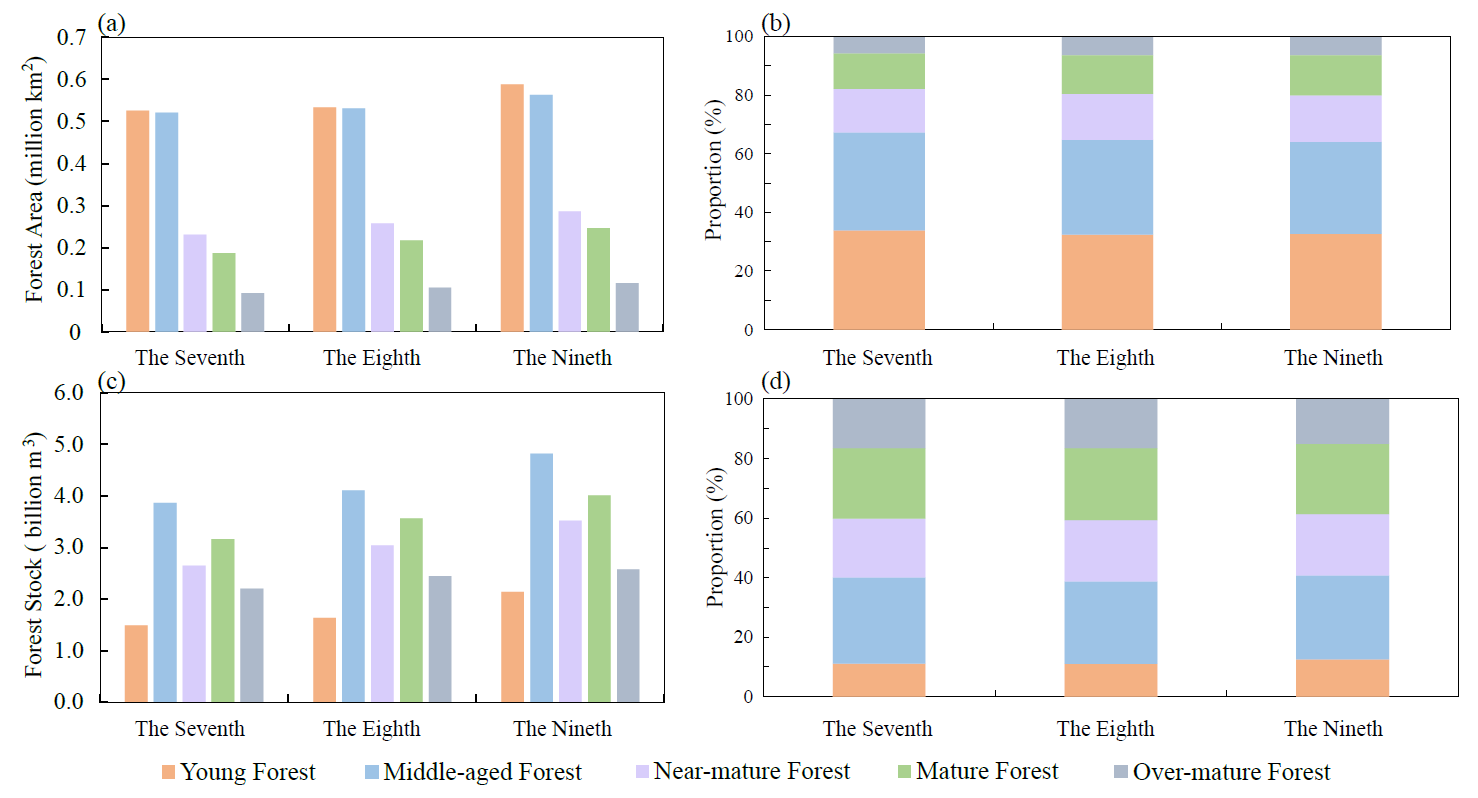


**Figure S3**. Forest area (a) and percentage of forest area (b) by five stand age groups from the 7th to 9th National Forest Inventories. Forest stock (c) and percentage of forest stock (d) by five stand age groups from the 7th to 9th National Forest Inventories.


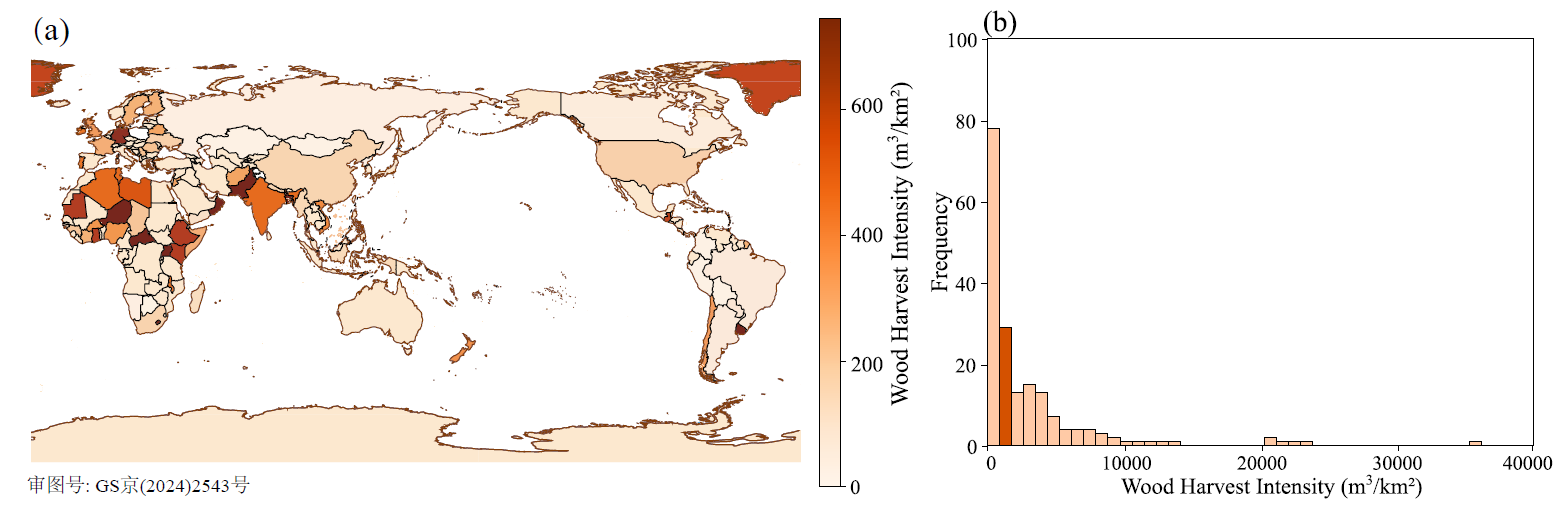


**Figure S4.** Global pattern of wood harvest intensity in 2020 (a) and frequency histogram (b).


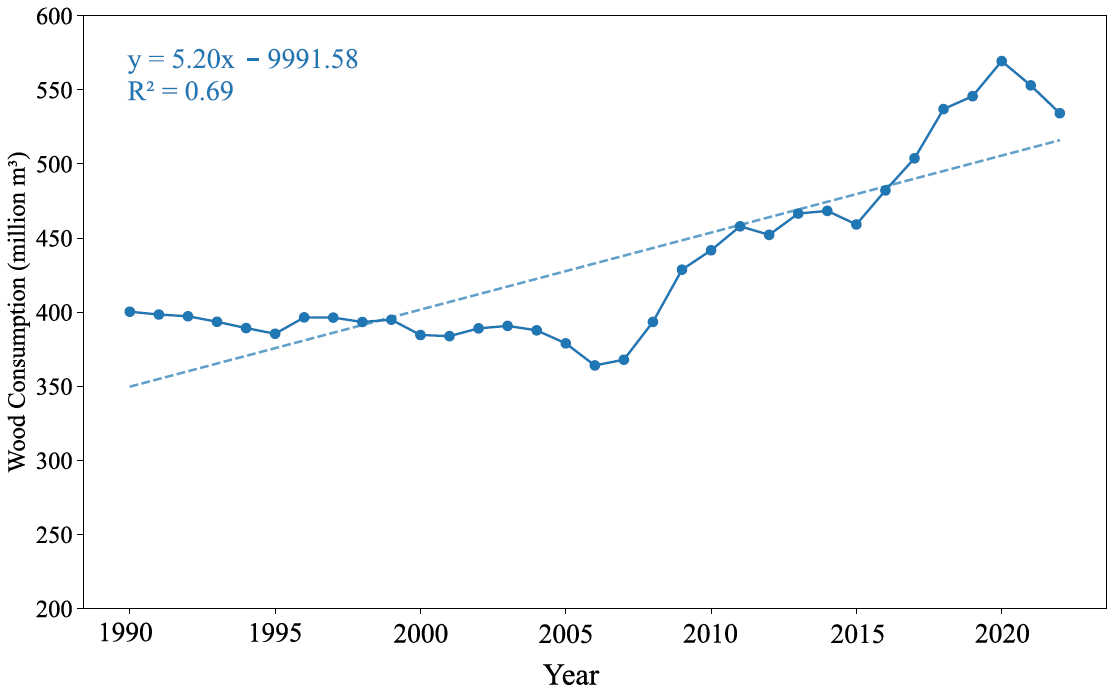


**Figure S5.** The long-term trend of wood consumption from 1990 to 2022 in China.


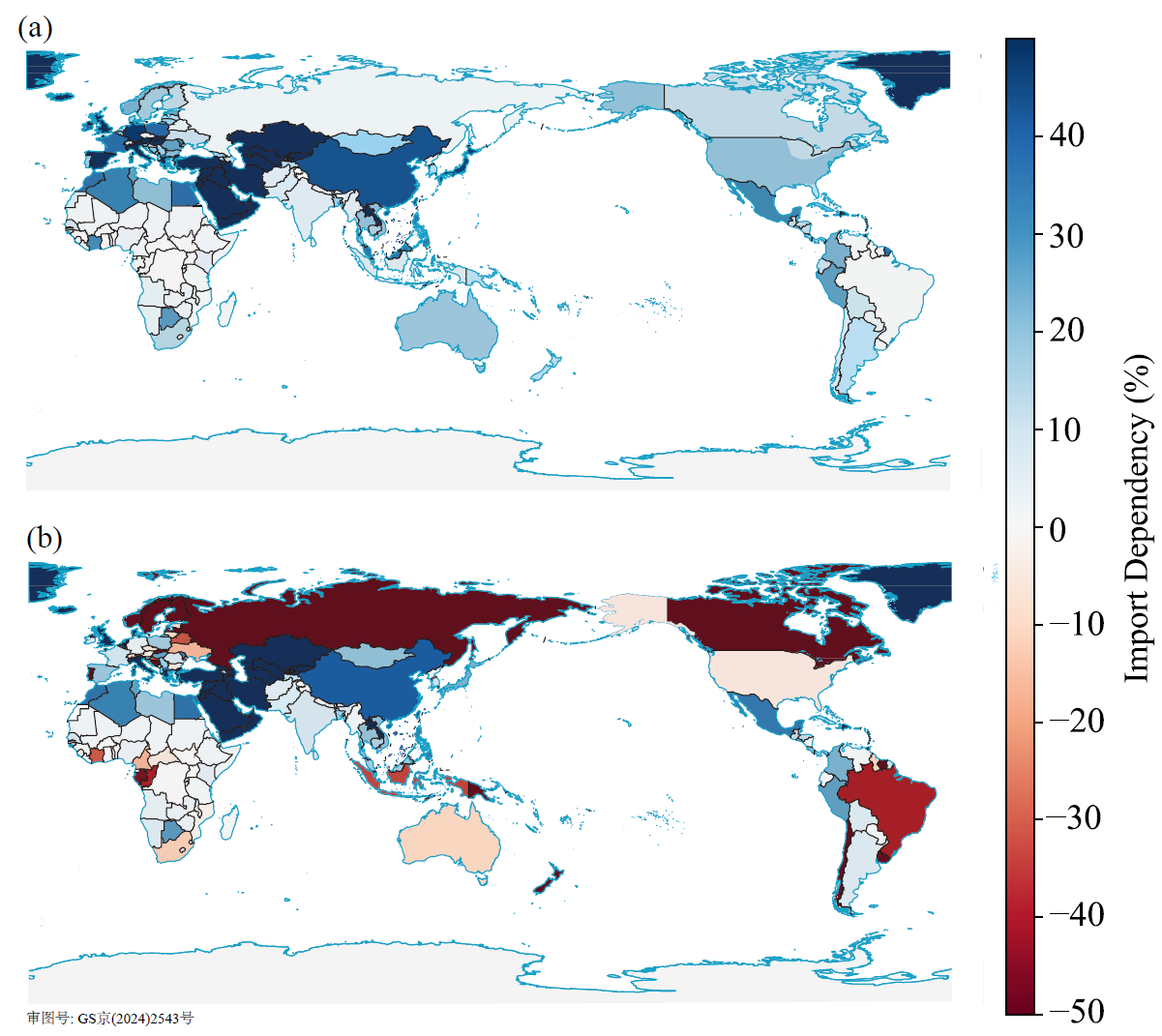


**Figure S6.** Global pattern of wood supply import dependency (a) and consumption import dependency (b) averaged from 2018 to 2022. The positive values indicate the net import countries of wood, and negative values indicate the net export countries.


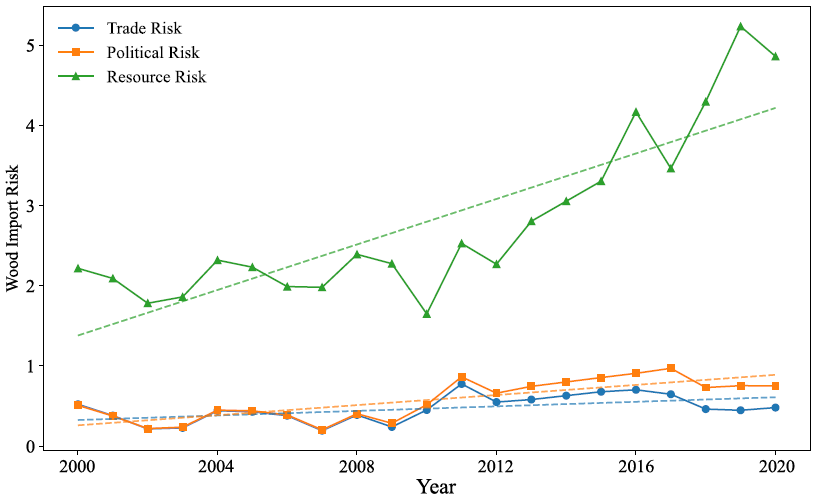


**Figure S7.** The long-term changes of three wood import risk indicators.


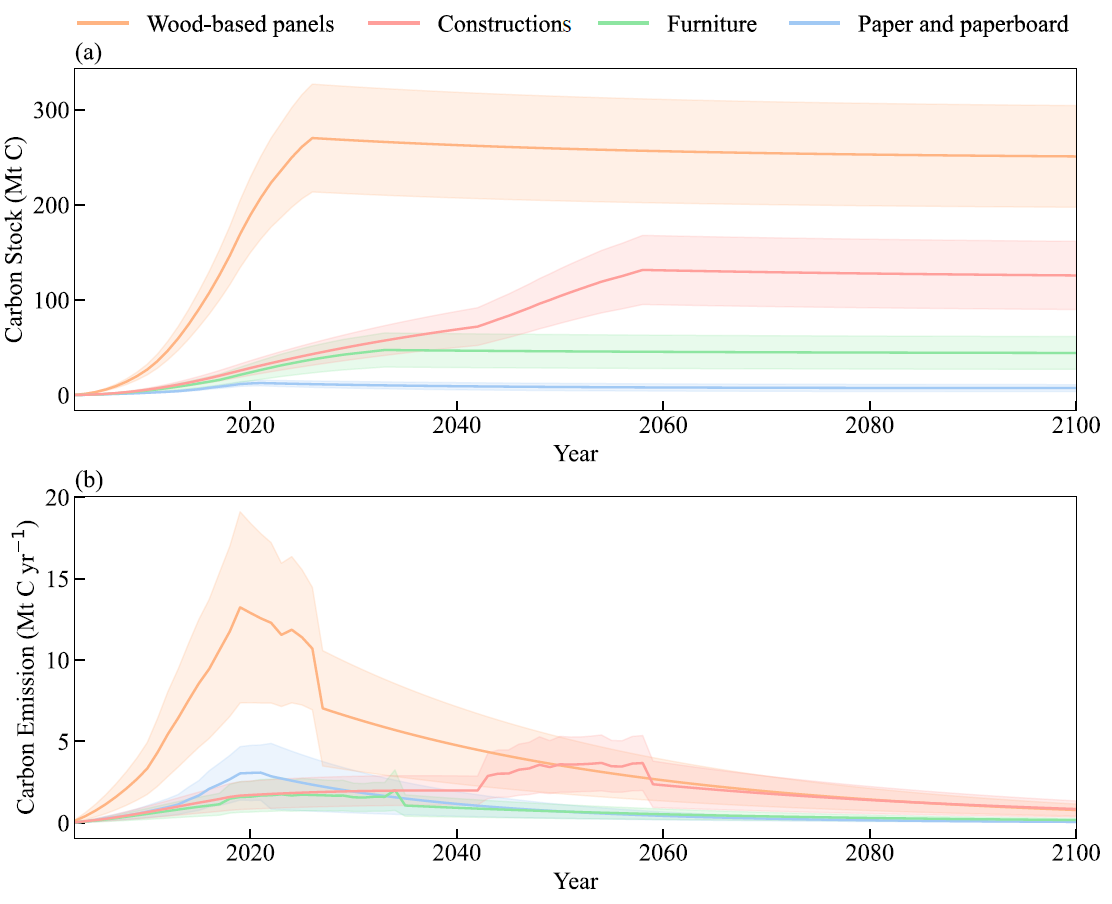


**Figure S8.** The accumulated carbon stock of harvested wood products (a) and annual carbon emission of harvested wood products (b).


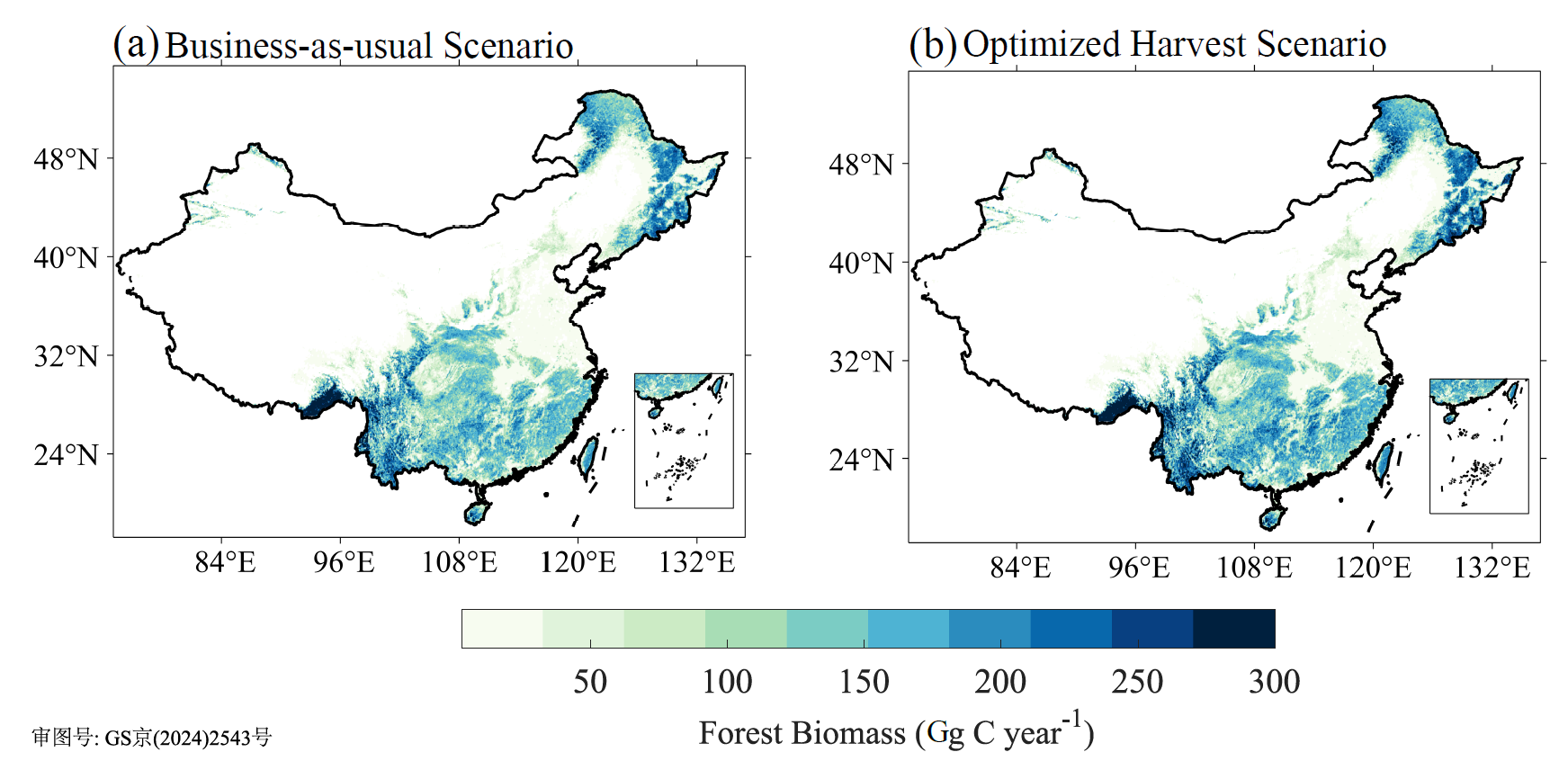


**Figure S9.** The spatial distributions of simulated forest biomass under business-as-usual scenario (a) and optimized harvest scenario (b) in 2060.


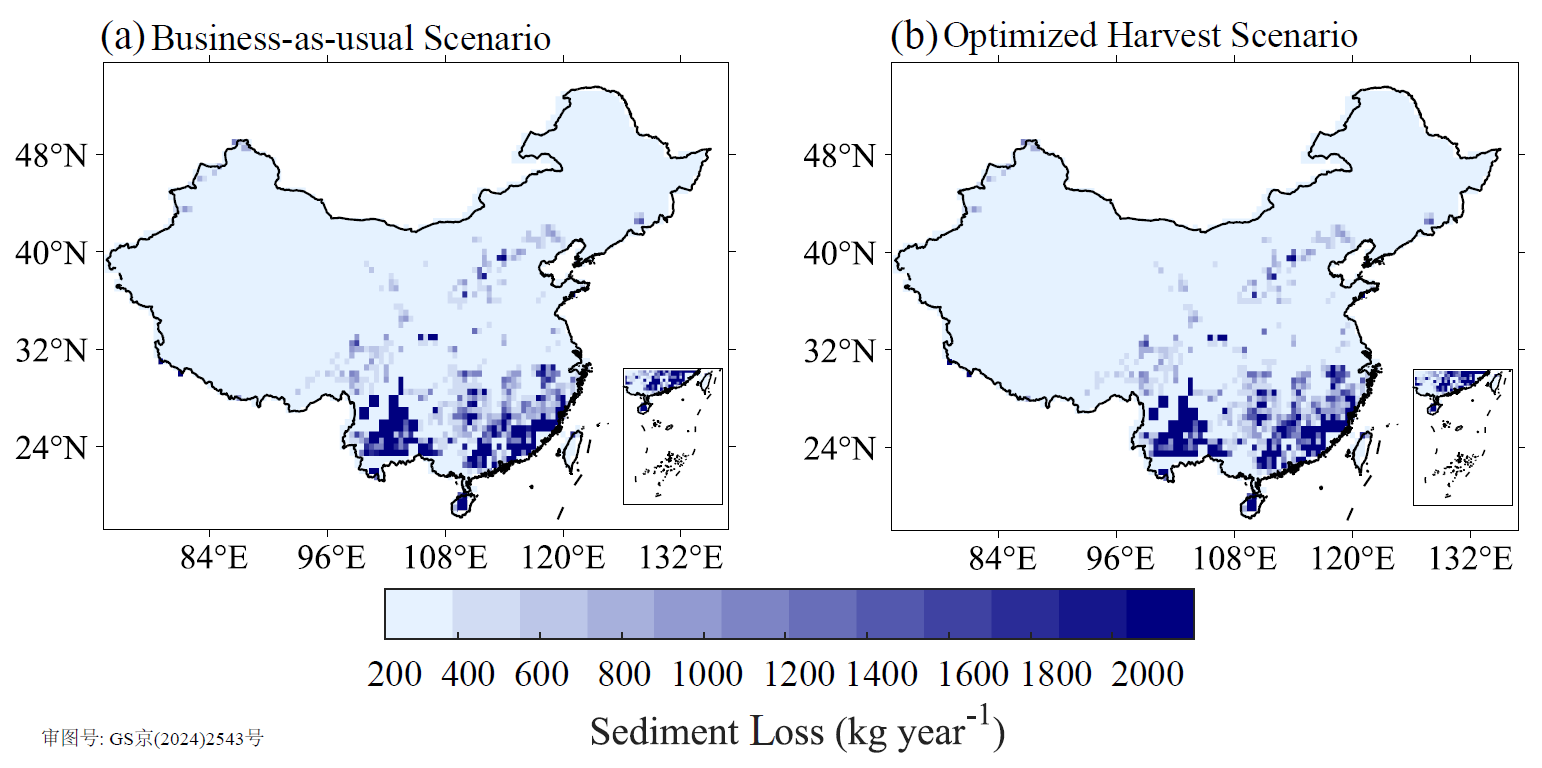


**Figure S10.** The spatial distributions of simulated soil erosion under business-as-usual scenario (a) and optimized harvest scenario (b) in 2060.

References

1. Tian M, Shi Y, Huang Y. Empirical analysis of the impact of China's economic development and timber product trade on wood consumption (in Chinese). *Scientia Silvae Sinicae*. 2016; **52**(09): 113-23.

2. Shi Y, Yu H, Tian M. A study on China's dependence on foreign trade of timber (in Chinese). *Forest Economics*. 2018; **40**(04): 25-32.

3. Huang W, Qiao S, Yang G. Demand elasticity and risk analysis of China's timber import (in Chinese). *Journal of Business Innovation*. 2020; **2**(2): 92-106.

4. Shang R, Chen J, Xu M *et al.* China’s current forest age structure will lead to weakened carbon sinks in the near future. *The Innovation*. 2023; **4**(6).

5. Tang X, Zhao X, Bai Y *et al.* Carbon pools in China’s terrestrial ecosystems: New estimates based on an intensive field survey. *Proceedings of the National Academy of Sciences*. 2018; **115**(16): 4021-26.

6. IPCC. *2013 Revised Supplementary Methods and Good Practice Guidance Arising from the Kyoto Protocol*. IPCC, Switzerland: 2014.

7. Krinner G, Viovy N, de Noblet‐Ducoudré N *et al.* A dynamic global vegetation model for studies of the coupled atmosphere‐biosphere system. *Global Biogeochemical Cycles*. 2005; **19**(1).

8. Guimberteau M, Perrier A, Laval K *et al.* A comprehensive approach to analyze discrepancies between land surface models and in-situ measurements: a case study over the US and Illinois with SECHIBA forced by NLDAS. *Hydrology and Earth System Sciences*. 2012; **16**(11): 3973-88.

9. Zhang H, Lauerwald R, Regnier P *et al.* Simulating erosion - induced soil and carbon delivery from uplands to rivers in a global land surface model. *Journal of Advances in Modeling Earth Systems*. 2020; **12**(11): e2020MS002121.

10. Liu Y, Gao X, Fu C. Estimating the Carbon Sequestration Potential of Forest Biomass in China Based on Forest Resource Inventory Data (in Chinese). *Acta Ecologica Sinica*. 2019; **39**(11): 4002-10.

11. Cheng K, Yang H, Guan H *et al.* Unveiling China’s natural and planted forest spatial–temporal dynamics from 1990 to 2020. *ISPRS Journal of Photogrammetry and Remote Sensing*. 2024; **209**: 37-50.

12. Cheng K, Chen Y, Xiang T *et al.* 2020 forest age map for China with 30 m resolution. *Earth System Science Data Discussions*. 2023; **2023**: 1-26.

13. Chen Y, Feng X, Fu B *et al.* Maps with 1 km resolution reveal increases in above-and belowground forest biomass carbon pools in China over the past 20 years. *Earth System Science Data*. 2023; **15**(2): 897-910.

14. Wang D, Ren P, Xia X *et al.* National forest carbon harvesting and allocation dataset for the period 2003 to 2018. *Earth Syst Sci Data*. 2024; **16**(5): 2465-81.
